# A Novel Method to Enhance the Mechanical Properties of Polyacrylonitrile Nanofiber Mats: An Experimental and Numerical Investigation

**DOI:** 10.3390/polym16070992

**Published:** 2024-04-04

**Authors:** Jaymin Vrajlal Sanchaniya, Inga Lasenko, Vishnu Vijayan, Hilary Smogor, Valters Gobins, Alaa Kobeissi, Dmitri Goljandin

**Affiliations:** 1 Institute of Mechanics and Mechanical Engineering, Faculty of Civil and Mechanical Engineering, Riga Technical University, 6B Kipsala Street, LV-1048 Riga, Latvia; inga.lasenko@rtu.lv (I.L.);; 2 NETZSCH Instrumenty, Halicka 9, 31-036 Krakow, Poland; hilary.smogor@netzsch.com; 3 Laboratory of Environmental Genetics, Institute of Biology, Faculty of Biology, Latvian University, Jelgavas Street 1, LV-1004 Riga, Latvia; valters.gobins@lu.lv; 4 Université de Technologie de Compiègne, Roberval (Mechanics, Energy and Electricity), Centre de Recherche Royallieu—CS 60319, 60203 Compiègne Cedex, France; alaa.kobeissi@utc.fr; 5 Department of Mechanical and Industrial Engineering, Tallinn University of Technology, Ehitajate Tee 5, 19086 Tallinn, Estonia; dmitri.goljandin@taltech.ee

**Keywords:** electrospinning, PAN nanofibers, PVA dopant, composites, TGA, DSC, FEM

## Abstract

This study addresses the challenge of enhancing the transverse mechanical properties of oriented polyacrylonitrile (PAN) nanofibers, which are known for their excellent longitudinal tensile strength, without significantly compromising their inherent porosity, which is essential for effective filtration. This study explores the effects of doping PAN nanofiber composites with varying concentrations of polyvinyl alcohol (PVA) (0.5%, 1%, and 2%), introduced into the PAN matrix via a dip-coating method. This approach ensured a random distribution of PVA within the nanofiber mat, aiming to leverage the synergistic interactions between PAN fibers and PVA to improve the composite’s overall performance. This synergy is primarily manifested in the structural and functional augmentation of the PAN nanofiber mats through localized PVA agglomerations, thin films between fibers, and coatings on the fibers themselves. Comprehensive evaluation techniques were employed, including scanning electron microscopy (SEM) for morphological insights; transverse and longitudinal mechanical testing; a thermogravimetric analysis (TGA) for thermal stability; and differential scanning calorimetry (DSC) for thermal behavior analyses. Additionally, a finite element method (FEM) analysis was conducted on a numerical simulation of the composite. Using our novel method, the results demonstrated that a minimal concentration of the PVA solution effectively preserved the porosity of the PAN matrix while significantly enhancing its mechanical strength. Moreover, the numerical simulations showed strong agreement with the experimental results, validating the effectiveness of PVA doping in enhancing the mechanical properties of PAN nanofiber mats without sacrificing their functional porosity.

## 1. Introduction

Electrospinning has gained prominence as an efficient and flexible approach to the fabrication of nanofiber mats, attracting considerable interest over the last two decades [1]. This method enables the production of nanofibrous structures through a straightforward single-step process, offering precise control over fiber diameters ranging from as small as ten nanometers to several hundred nanometers. Electrospun polyacrylonitrile (PAN) nanofibers serve as a fundamental base for continuous carbon nanofibers [2], optical and sensor materials [3], nanocomposites [4], tissue scaffolds [5,6], wound healing [7,8], drug delivery systems [9], filtration solutions [10], protective gear [11,12], and intelligent textiles [13].

The mechanical efficacy of electrospun nanofibers is critical in determining the structural attributes of a nanofiber mat, such as its fiber diameter [14], fiber orientation [15], and mat porosity [15,16]. In particular, a high degree of nanofiber alignment correlates with enhanced stiffness and improved mechanical properties. However, when nanofibers are closely aligned, their transverse mechanical stiffness largely depends on the inter-fiber bonding within the mat [17], due to the absence of fibers oriented in the transverse direction to bear loads.

To ameliorate a nanofiber mat’s mechanical properties, it is crucial to either strengthen these inter-fiber bonds or introduce additional structural support to manage the load in the transverse direction. A significant challenge with enhancing these nanoscale bonds is the selection of an appropriate polymer that can create effective bonding without adversely affecting the properties of the PAN nanofibers. Common industrial solvents, such as DMF and formic acid, could potentially impact their properties, with residual solvent potentially altering mechanical strength and thermal stability due to effects on fiber flexibility [18,19,20]. Moreover, the polymer solution must be sufficiently low in viscosity to penetrate and bond with the nanofibers while preserving their porous structure. Polyvinyl alcohol (PVA), which is water-soluble and capable of forming a polymer solution with a minimal impact on viscosity at concentrations ranging from 0.5% to 2.0%, addresses these requirements effectively.

An exhaustive review by Nauman et al. [21] has analyzed various techniques used to enhance the mechanical properties of nanofiber mats, including strategies such as chemical cross-linking [22], hot stretching [23], hot pressing [24], annealing [25], drawing [26], and solvent welding [27]. Each of these methods presents its own set of limitations, and not all are applicable to the diverse range of polymers employed in nanofiber fabrication. The previous work of the authors [25] highlighted the significant enhancement in the mechanical properties of oriented PAN nanofibers after their annealing at 70 degrees Celsius. It was observed that annealing beyond this temperature led to a decrease in the nanofibers’ mechanical properties, which was attributed to the evaporation of the solvents used at higher temperatures. Additionally, research by Stachewicz et al. [28] demonstrated the impregnation of nylon (PA6) nanofibers into a high-concentration polymer solution, which, while improving the mechanical properties of the PA6 nanofiber mat, compromised its porosity, because the PVA solution filled the gaps within the mat, transforming it into a composite polymeric film. Meanwhile, Tang et al. [29] have illustrated that a dip-coating method is predominantly utilized to fabricate functional fibrous materials for applications such as self-cleaning surfaces [30], conductive textiles [31], oil–water separation [32], energy storage [33], and photonic crystals [34].

A significant gap in the literature emerges from the absence of a direct method to fortify the strength of PAN nanofiber mats while preserving their essential porosity. Addressing this gap, the authors have endeavored to create PAN nanofiber mats doped with PVA, aiming to achieve both these goals. This innovative approach seeks to leverage the benefits of PVA doping, applied through a dip-coating method, to provide a balanced enhancement of structural integrity and functional porosity, thereby presenting a promising avenue for advancing the utility and application of PAN nanofiber composites.

This study seeks to develop the efficacy of a dip-coating technique designed to enhance the structural integrity of nanofiber mats through the application of water-soluble, low-concentration polyvinyl alcohol (PVA). Utilizing PVA concentrations ranging from 0.5% to 2.0% has been shown to minimally impact the viscosity of the polymer solution, enabling the successful creation of polyacrylonitrile (PAN) nanofiber composites doped with PVA. A thorough suite of analyses was conducted on these PAN composites to discover the degree to which their structural support and performance increased. These analyses included evaluations of their thermal properties, scanning electron microscopy (SEM) for a detailed morphological examination, thermal tests, and mechanical tests. The results of these assessments provided significant insight into the enhancement of the PAN composites. Furthermore, the development and validation of a finite element method (FEM) model against the experimental findings served to reinforce the effectiveness of this PVA doping process. This validation underscores the ability of low-concentration PVA to significantly bolster the structural integrity of PAN nanofiber mats, highlighting the potential of this method in advancing the functional applications of nanofiber composites.

## 2. Materials and Methods

### 2.1. Materials

To develop a polyacrylonitrile (PAN) nanofiber mat doped with polyvinyl alcohol (PVA), a fundamental step is producing the electrospun nanofibers. This process utilized a PAN powder dissolved in N,N-dimethylformamide (DMF), along with a separately prepared solution of PVA dissolved in distilled water. The specific materials used in this study were carefully selected for their purity and molecular weight to ensure consistency and reliability in the electrospinning process and the subsequent doping procedure. The PAN used had an average molecular weight (MW) of 150,000 (typical) and a CAS number of 25014-41-9. The solvent used, DMF, is an ACS reagent (solvent) with a CAS number of 68-12-2 and was chosen for its high purity, 99.8%. For PVA, a material with an average MW of 89,000–98,000 and a hydrolysis level of 99+% was selected, identified by the CAS number 9002-89-5. All these chemicals were sourced from Sigma-Aldrich Chemicals (Merck KGaA, Steinheim, Germany), ensuring that they were high-quality and standardized materials for the fabrication of doped PAN nanofiber mats.

### 2.2. Fabrication of PAN Nanofiber Mat Doped with PVA

The PAN nanofibers were synthesized using an electrospinning setup featuring a rotating drum collector, following the protocol previously described by the authors [15,25]. To prepare the PAN solution, a 10% (wt./wt.) PAN powder was thoroughly dissolved in N,N-dimethylformamide (DMF) under continuous stirring at 80 °C and 1000 rpm for 4 h, using a magnetic stirrer. The solution was then electrospun using a 10 mL Luer lock syringe equipped with an 18-gauge needle, which has an outer diameter of 1.27 ± 0.01 mm and an inner diameter of 0.838 ± 0.01 mm. The electrospinning process was carried out under specific conditions: a voltage of 20 kV was applied, the solution was fed at a rate of 1 mL/h, the rotating drum was operated at a speed of 2100 rpm, and the distance between the syringe needle and the collector drum was kept constant at 18 cm.

Following the successful electrospinning of PAN nanofibers, these mats were then doped with PVA using a dip-coating method. To achieve this, three different PVA solutions were prepared, each with a different concentration: 0.5%, 1%, and 2% wt./wt. These solutions were created by dissolving the appropriate amount of PVA powder in distilled water, followed by stirring at 400 rpm with a magnetic stirrer and heating to 95 °C for one hour. This process ensured that the PVA was completely dissolved and the solution was homogenous.

Once the PVA solutions reached room temperature, the PAN nanofiber mats were cut into specified sizes to act as specimens. These mats were then immersed in the PVA solutions for one minute, ensuring that the voids within the nanofiber mats were filled with the PVA solution. After coating, the mats were left to dry for 72 h. This drying period allowed the solvent to evaporate completely, leaving behind a PAN nanofiber mat uniformly doped with PVA. The dip-coating process is a critical step in enhancing the mechanical properties of the nanofiber mats while maintaining their inherent porosity, a key aspect to their application in filtration and other areas.

**Figure 1:**
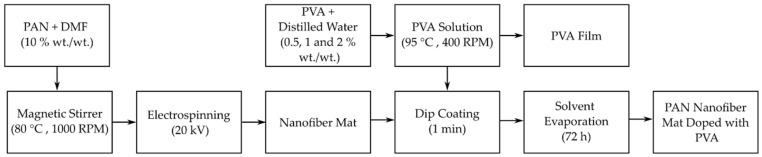
Fabrication process of PVA-doped PAN nanofiber mats.

The entire process of fabricating the PVA-doped PAN nanofiber mat, from the preparation of the PAN solution to its final dip-coating in PVA, is illustrated in Figure 1. This figure visualizes the step-by-step methodology, highlighting the crucial stages in the production of enhanced nanofiber mats that are capable of offering improved performance in various applications.

This detailed procedure underscores the methodical approach taken to develop PVA-doped PAN nanofiber mats that utilize the strengths of both PAN and PVA to create composites with superior structural integrity and functionality.

After dip-coating the PAN nanofiber mats, the remaining PVA solution was carefully poured into a Petri dish to form a thin film. This procedure was meticulously carried out to ensure a uniform spread of the solution across the base of the plate, facilitating the formation of an even and consistent film upon drying. The Petri dishes containing the PVA solutions were then left undisturbed at room temperature, allowing the solvent to evaporate naturally and the PVA to solidify into a film.

The creation of this PVA film served a dual purpose. First, it provided a pure PVA sample that could be directly analyzed to understand the baseline properties of the polymer used for doping. Second, and more importantly, it allowed for a direct comparison between the thermal and mechanical properties of the pure PVA film and those of the PVA-doped PAN nanocomposite mats. When these properties were found, we could isolate the effects of PVA doping on the nanofibers, distinguishing the intrinsic properties of the PVA from the synergistic or composite effects observed in the doped nanofiber mats.

This comparison is essential for validating the effectiveness of PVA doping in enhancing the properties of PAN nanofiber mats. It also provides insight into the compatibility and interaction between PAN and polyvinyl alcohol (PVA), contributing to our deeper understanding of the behavior of this composite material under various conditions. This comparative analysis is crucial for tailoring the properties of composites to specific applications by adjusting the type and concentration of the doping agents used in their production.

### 2.3. Porosity Test

To determine the porosity of the nanofiber mats, a density comparison method was used, as mentioned in [15], which calculates the percentage of their porosity (P) using the following equation:

(1) $$P\left(\%\right)=\left(1-\frac{M}{M_{d}}\right)\times 100,$$

where P(%) represents the porosity percentage, M is the measured mass of the nanofiber mat in units of µg, and M_d_ is the theoretical mass that the specimen would possess if it were fully dense. The value of M_d_ is calculated using the formula V × ρ, where V represents the volume (cm^3^) of the specimen and ρ is its density (1.184 g/cm^3^).

In the context of the PVA-doped PAN nanofiber mats, an increase in the mass of the specimens was observed after doping, indicative of the incorporation of PVA into the mats. This mass change, alongside the newly calculated volume of the doped mats, facilitated the determination of the percentage of PVA (at density: 1.08 g/cm^3^) present within the specimens, providing information on how doping affects the porosity of the nanofiber mats. Mass measurements were performed using a high-precision KERN ABT 5NM scale, which boasts a maximum capacity of 100 g and a precision of 0.000001 g, thus ensuring accurate and reliable results. The scale’s detailed calibration specifications, including its serial number (WB22G0101) and calibration certificate (B61-389-2023-03/1, dated 24 March 2023), underscore the rigorous approach adopted for these measurements.

### 2.4. Morphology Observation

The morphological characteristics of the PVA-doped PAN nanofiber mats were examined using a Hitachi TM300 tabletop scanning electron microscope (SEM), as mentioned in [4,15,35]. The SEM was operated at a magnification of 1500×, under a vacuum of 10^−2^ Torr, and the samples were ion-coated with gold (Au) to a thickness of 150 Å. The average fiber diameter was evaluated using ImageJ software (version 1.54 h) [36,37,38], with measurements taken from 100 nanofibers randomly selected from three different SEM images. This process not only allowed for the assessment of the uniformity and distribution of fiber diameters within the doped mats but also facilitated a detailed comparison of the morphological changes seen after doping. An enhancement of image contrast was employed to better visualize and measure the fibers, providing a comprehensive understanding of the microstructural implications of PVA doping PAN nanofiber mats.

### 2.5. Tensile Test

For the evaluation of the tensile strength of the PAN nanofiber mats, PVA films, and the PVA-doped PAN nanofiber mats, meticulous preparation and testing protocols were adhered to, and a Mecmesin Multi-Test 2.5-i tensile tester equipped with sensors capable of handling forces up to 25 N and 250 N was used, supplied by PPT Group UK Ltd. Before testing, samples were conditioned as stipulated by [39] maintained at a stable temperature of 21 ± 1 °C, a relative air humidity of 60%, and an atmospheric pressure of 760 mm Hg—to ensure that the properties of the materials were not altered by external variables.

The test samples were 50 mm in length and 10 mm in width, conforming to the ASTM D882-18 standard for the mechanical testing of thin plastic sheeting. This standardization was critical to ensure the repeatability and reliability of our tensile test results. To accurately gauge the thickness of each sample, a highly precise Digimatic micrometer (MDC-25PX, Mitutoyo, Japan) with the serial No. 71912410 was used, which offers measurement capabilities in the range of 0 to 25 mm, ensuring that each measurement contributed to the precision of the stress calculations.

Specimens were prepared for testing in both the longitudinal and transverse orientations relative to their fiber direction to comprehensively understand their mechanical behavior under tensions from all directions. For this purpose, a unique mounting technique was employed: specimens were affixed to a custom-cut 50 mm × 40 mm paper template featuring an internal cutout of 30 mm × 20 mm using double-sided thin Scotch tape (3M Scotch Magic Tape, Riga, Latvia.), as mentioned in [25,35]. This method facilitated the secure attachment of the nanofiber mat specimens to the tensile tester, while allowing for easy cutting of the paper template sides just before the test, thus avoiding any potential interference with the material’s tensile properties.

### 2.6. Thermal Test

To evaluate the thermal stability and transitions of the PAN nanofiber mats doped with PVA, we performed a comprehensive thermal analysis using both a thermogravimetric analysis (TGA) and differential scanning calorimetry (DSC).

For the TGA, a NETZSCH TG 209 F1 Libra® thermomicrobalance from Selb, Germany, was utilized. Small samples, each weighing between 5 and 6 mg, were placed in aluminum oxide (Al_2_O_3_) crucibles. This analysis involved increasing the temperature from 20 °C to 800 °C at a consistent rate of 10 °C/min. Throughout this process, the samples were maintained in an inert nitrogen atmosphere, ensuring that no oxidative degradation occurred, with the nitrogen flow rate set at 30 mL/min, as mentioned in [25]. This test aimed to pinpoint the thermal decomposition temperatures and evaluate the thermal stability of the doped nanofiber mats.

Following the TGA, DSC tests were performed in alignment with the ASTM E1356 standards [40]. These were performed using a DSC 214 Polyma differential scanning calorimeter, also provided by NETZSCH. For the DSC analysis, PAN nanofiber mats, both undoped and doped with PVA, along with a pure PVA film for comparison, were placed in the DSC crucible. This analysis entailed a two-cycle heating process: initial heating from −50 °C to 150 °C to eliminate any impurities or moisture, followed by cooling to −50 °C, and subsequent reheating to 150 °C. This procedure was designed to observe the glass transition temperature, as well as other thermal transitions. Each heating and cooling phase was separated by a one-minute isothermal step to stabilize the temperature across the sample, ensuring accurate and reproducible results, as previously mentioned by the authors [25].

This thermal analysis provided critical insights into the material properties of these PVA-doped PAN nanofiber mats by determining their thermal stability and identifying their thermal transitions. This will help us better understand the effects of PVA doping on the structural and chemical integrity of PAN nanofibers under varying thermal conditions, which is vital for predicting the performance and application range of these nanocomposite materials in environments subject to thermal stress.

## 3. Finite Element Model

**Figure 2:**
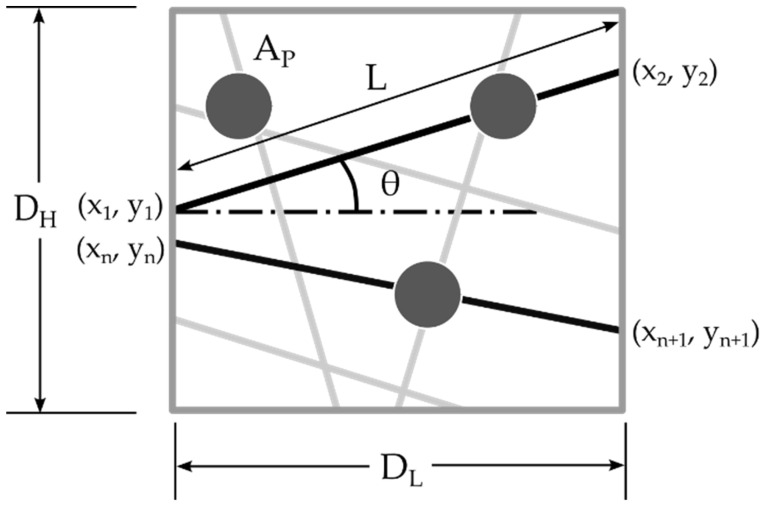
Schematic representation of the geometric parameters of nanofibers doped with PVA within an established domain.

The development of a finite element (FE) model for the PVA-doped PAN nanofiber mats was based on methodologies previously established by the authors [15]. This model incorporates geometric parameters crucial for accurately representing the structural characteristics of the nanofibers after doping them with PVA. Figure 2 shows that the key parameters include domain length (D_L_) and height (D_H_), which define the spatial confines of the nanofiber mat model. Within this area, the length (L) of the nanofibers, their diameter, and the coordinates of each fiber’s endpoints (x_1_, y1 and x_2_, y_2_) were meticulously established to reflect the controlled porosity of the mat. The increase in the mass of the nanofiber mat after doping was represented by the area of PVA (A_P_) relative to the domain area, illustrating the concentration of the dopant within the mat.

In the methodology for generating the FE model, assumptions consistent with those made in previously developed models [15] were applied. It was assumed that the PVA, when introduced as a dopant, would only occupy areas at the fiber intersections, effectively modeling the real-life distribution of the doping material within the nanofiber mat. This assumption is critical for accurately simulating the influence of PVA doping on the mechanical properties of the nanofiber mat, as it reflects the targeted enhancement of inter-fiber bonding without altering the inherent structural configuration of the undoped sections of the mat.

**Figure 3:**
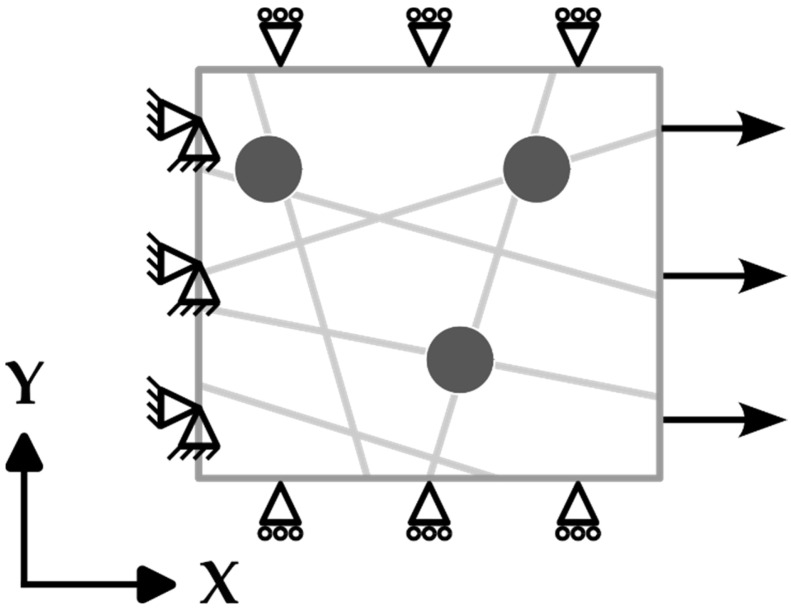
Boundary conditions applied to observe the normal stress response of the samples to displacement.

This model was designed to simulate the mechanical behavior of nanofiber mats under tensile loading. Specific boundary conditions (as shown in Figure 3) were applied to capture their response to displacement, particularly focusing on the reaction force (RF) in the X axis direction. The displacement in the X, Y, and Z axes is considered as *U*_1_, *U*_2_, and *U*_3_, respectively. A displacement of up to 5% of the domain length was imposed on one of the ends of each of the fibers, with constraints to prevent movement applied in the Y and Z directions (*U*_2_ = *U*_3_ = 0), ensuring a realistic simulation of the material’s stretching behavior. The opposite ends of the fibers were fixed in all directions (*U*_1_ = *U*_2_ = *U*_3_ = 0) to mimic the anchoring effect achieved in a tensile test, but rotational freedom around the Z axis was allowed to account for the angular adjustments resulting from the applied displacement. The sides perpendicular to the direction of displacement were set to move in sync with the direction of displacement, avoiding any sliding towards each other, and the upper and lower boundaries were assigned conditions to restrict their movement in the Y and Z directions (*U*_2_ = *U*_3_ = 0).

In the finite element analysis (FEA) conducted in this study, the structural components of the nanofiber mats, specifically the fibers themselves, were modeled using linear beam elements, identified as B31 in the Abaqus FEA software package (2022). The dopant, in this case polyvinyl alcohol (PVA), was modeled using shell elements (S4R). Shell elements are a versatile choice for representing thin layers that have a definite thickness, providing an accurate approximation of the PVA doping layer enveloping the PAN nanofibers.

To address the inherent variability in generating fibers with specific orientations, porosity levels, and dopant sizes—which can lead to diverse structural outcomes from the same set of parameters—multiple simulations were conducted for each parameter combination. Conducting at least five simulations per set of parameters ensured that the derived elastic response was reliable and representative of the material’s behavior, thereby enhancing the validity of the FE model’s predictions regarding the doped nanofiber mats’ performance under tensile stress.

To accurately simulate the physical connections between the nanofibers and the dopant, the beam and shell elements were interconnected using tie joints. This method of connection permits rotational movement around the joint, reflecting the potential for relative movement between the fibers and the dopant under mechanical loading. By employing tie joints, the model can more accurately reflect the complex mechanical behavior of the nanocomposite material [41], especially under tensile stresses where such rotational freedoms may influence the overall responses of the material.

## 4. Results and Discussion

### 4.1. Morphology

**Figure 4:**
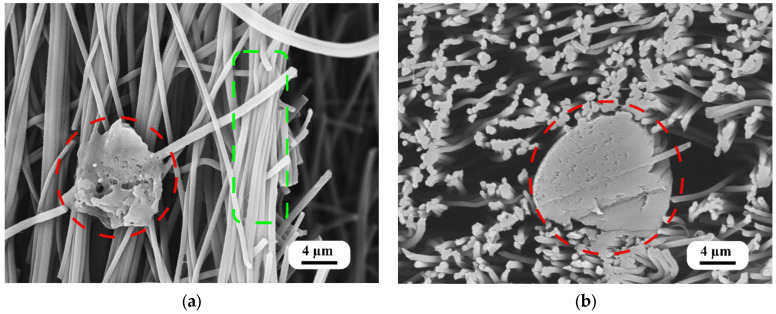
SEM images of a PAN nanofiber mat doped with a 2.0% PVA solution: (**a**) localized agglomerations of PVA within the nanofiber mat (red) and coating on fibers (green); (**b**) cross-sectional image of the localized agglomerations of PVA.

The morphology of the PVA-doped PAN nanofiber mats and their cross-sections, as illustrated in Figure 4, elucidates the multifaceted impact of the quick dip-coating process. This figure displays the nanofiber mats treated with a 2.0% PVA solution, where three distinct effects are observed: localized agglomerations of PVA within the nanofiber mat, the formation of thin films of PVA between PAN nanofibers, and a coating of PVA on the surface of the PAN nanofibers. These morphological variations are critical for bolstering the structural integrity of the mats by introducing additional points of interaction between the fibers, thus improving the overall mechanical properties of the material.

The presence of localized PVA agglomerations and the PVA coating on the fibers signify a strategic modification in the morphology of the fiber mat, potentially facilitating improved load distribution and inter-fiber bonding. Moreover, the thin nanoscale PVA films between the nanofibers likely serve to further distribute mechanical stress across the mat, enhancing its overall strength and resilience.

The obtained morphological changes via dip-coating are in line with our expectations, confirming the process’s ability to create thin coatings, films, and agglomerations of PVA within a PAN nanofiber mat. These results align with observations previously made by researchers [29,42], where dip-coating was predicted to modify the surface and internal structure of nanofiber mats.

The diameter of the nanofibers was measured to be 609 ± 43 nm, highlighting the uniformity and precision achieved through the electrospinning process. The results obtained are similar to a prior study of electrospinning performed with the same parameters [15]. This measurement reflects the controlled conditions under which the PAN nanofibers were fabricated, ensuring consistent fiber sizes across the mat. Such uniformity is crucial for applications that rely on specific surface area and porosity characteristics, as they influence the material’s mechanical and filtration properties.

These findings reveal the nuanced interaction between PVA and PAN within the nanofiber mats and affirm the efficacy of the dip-coating process in modifying the mats’ structural properties. The analysis of these morphological characteristics underlines the suitability of PVA-doped PAN nanofiber mats for applications in which materials with superior mechanical strength and structural integrity are required.

### 4.2. Porosity

The quantification of the porosity of both pure and PVA-doped PAN nanofiber mats through the density comparison method reveals a notable trend: doping with PVA leads to a systematic decrease in porosity. Initially, the porosity of the pure PAN nanofiber mat was 72 ± 0.5%. This value represents the inherent open structure and large surface area typically desired in applications such as filtration and tissue engineering. However, upon doping with PVA at varying concentrations, a gradual decrease in porosity was observed. Specifically, doping with a 0.5% PVA solution reduced the mat’s porosity to 69 ± 0.5%, which further dipped to 67 ± 0.4% with 1% PVA and reached 63 ± 0.7% when a 2% PVA concentration was used. Table 1 presents a summary of the porosity measurements for both the pure and PVA dip-coated PAN nanofiber mats.

The reduction in porosity with the increasing PVA concentration can be attributed to several factors. Primarily, the PVA doping process, specifically dip-coating, introduces additional PVA into the spaces between PAN nanofibers, effectively filling the gaps and creating a denser structure. This filling effect is supported by the morphological changes observed (Figure 4a,b), such as the formation of thin PVA films and agglomerations within the nanofiber mat, which contribute to its decrease in porosity. Additionally, solvent evaporation during the PVA solidification process might contribute to the compaction of the nanofiber mat, further reducing its porosity.

This trend has significant implications for the functional properties of nanofiber mats. Although a decrease in porosity might affect the mat’s filtration efficiency or tissue scaffold performance due to reduced permeability, it could also enhance its mechanical strength and structural integrity, as suggested by the morphological changes observed. Therefore, the balance between porosity and material density must be carefully considered in the context of the intended application of the PVA-doped PAN nanofiber mat. This understanding allows for the targeted adjustment of PVA concentrations during the doping process to achieve desired material properties, paving the way for the development of composites with tailored performance characteristics.

### 4.3. Mechanical Properties

**Figure 5:**
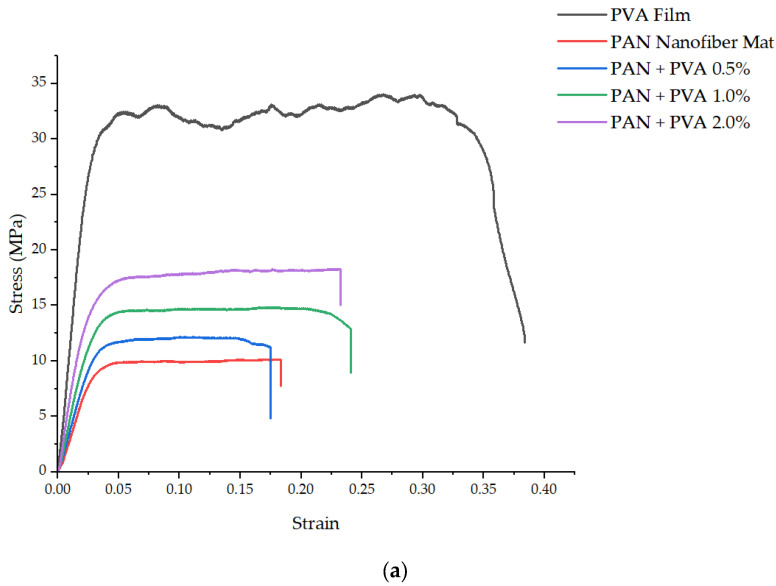

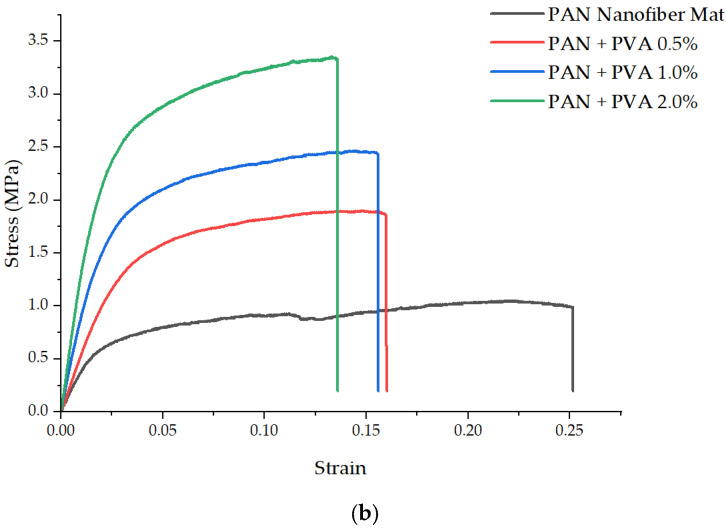
Representative stress–strain graphs: (**a**) longitudinal direction, (**b**) transverse direction.

Figure 5a,b present the stress–strain curves obtained from the tensile testing of the PVA films and the PAN nanofiber mats, tested in both transverse and longitudinal directions, including the mats doped with varying concentrations of PVA. The PVA film exhibited an elastic modulus of 1254 ± 57 MPa and an ultimate tensile strength of 34 ± 2.4 MPa, with a notably high plasticity and elongation at break of 0.38 ± 0.03. Consistency in the thickness of both the undoped and PVA-doped PAN nanofiber mats was observed, suggesting that the low percentage of dopant used did not significantly alter their thickness.

The elastic modulus of the pure PAN nanofiber mat was measured as 383 ± 34 MPa and its ultimate tensile strength was 9.9 ± 0.8 MPa, with an elongation at break of 0.18 ± 0.02. On the other hand, in the transverse direction, its elastic modulus was significantly lower, 47 ± 6 MPa, and its ultimate tensile strength was 1.13 ± 0.15 MPa, with an elongation at break of 0.21 ± 0.02.

Doping the PAN nanofiber mats with PVA led to an improvement in their mechanical properties in both the transverse and longitudinal directions. Specifically, in the longitudinal direction, the elastic modulus increased to 475 ± 42 MPa, 538 ± 29 MPa, and 683 MPa for 0.5%, 1%, and 2% PVA, respectively. The transverse direction saw increases in the elastic modulus to 65 ± 4 MPa, 81 ± 6 MPa, and 122 ± 12 MPa for 0.5%, 1%, and 2% PVA, respectively. Their ultimate tensile strength in the longitudinal direction also increased with the concentration of PVA to: 12.1 ± 1.1 MPa, 14.9 ± 1 MPa, and 18.25 MPa for 0.5%, 1%, and 2% PVA, respectively. Similarly, in the transverse direction, their ultimate tensile strength increased to 1.87 ± 0.11 MPa, 2.5 ± 0.1 MPa, and 3.4 ± 0.24 MPa for 0.5%, 1%, and 2% PVA, respectively. Table 2 provides a comparative overview of the mechanical properties across PVA films, pure PAN nanofiber mats, and PVA-doped PAN nanofiber mats.

The mechanical properties we observed for the independent PVA films align with the findings of other researchers [43,44,45,46], and the properties of the PAN nanofiber mat are consistent with those previously observed in a mat made by the authors under the same electrospinning parameters [15]. The enhancement of the mechanical properties of the doped nanofiber mats underscores the effective structural support provided by PVA, both as an inter-fiber bond and as a coating on the fibers. In particular, the introduction of a 2% PVA dopant led to an 84.34% increase in the ultimate tensile strength and a 78.33% increase in the elastic modulus of the PAN nanofiber mat in the longitudinal direction. In the transverse direction, these properties saw even more significant increases, with the ultimate tensile strength rising by 200% and the elastic modulus by 159.5%.

The observed improvements in mechanical properties, particularly in the transverse direction, can be attributed to the role of PVA in enhancing the bonds between the oriented fibers, where the load-bearing capacity of a nanofiber mat is typically limited by its fiber bonds. The presence of PVA as a dopant not only strengthens these bonds but also changes the mat’s failure mode to fiber shear rather than debonding, thereby significantly enhancing its overall mechanical strength.

### 4.4. Thermal Properties

**Figure 6:**
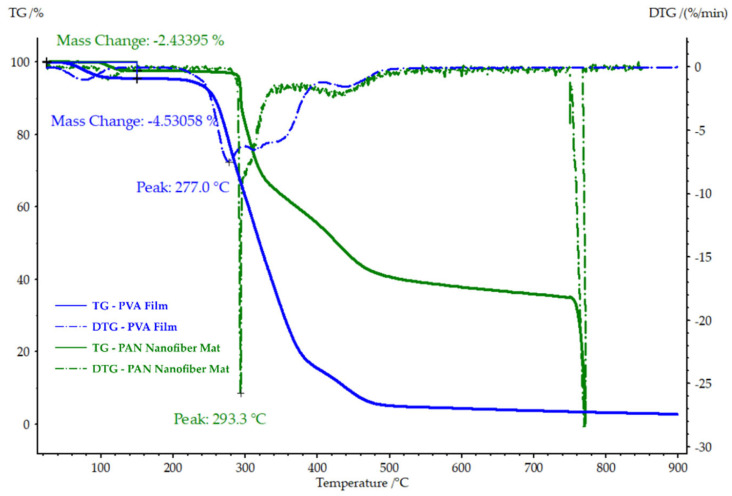
TGA and DTG of the pure PAN nanofiber mat and PVA film, highlighting their thermal degradation points and initial mass loss due to solvent evaporation.

The thermogravimetric analysis (TGA) and derivative thermogravimetry (DTG) graphs depicted in Figure 6 provide insight into the thermal stability and degradation behavior of the pure PAN nanofiber mat and the PVA film at varying temperatures. The DTG graph reveals a notable peak drop at 293.3 °C for the PAN nanofiber mat and at 277.0 °C for the PVA film, indicating the primary degradation temperatures of each material. Furthermore, the mass loss up to 150 °C was 2.43% for the PAN nanofiber mat and 4.53% for the PVA film, suggesting initial solvent evaporation or moisture loss from the samples.

This early mass loss in the PAN nanofiber mat is consistent with previous findings by the authors [25], who attributed this reduction to the evaporation of residual solvent. A similar pattern of early stage mass loss was observed for the PVA film, aligning with observations made in other studies [47,48], where PVA exhibited comparable behavior.

**Figure 7:**
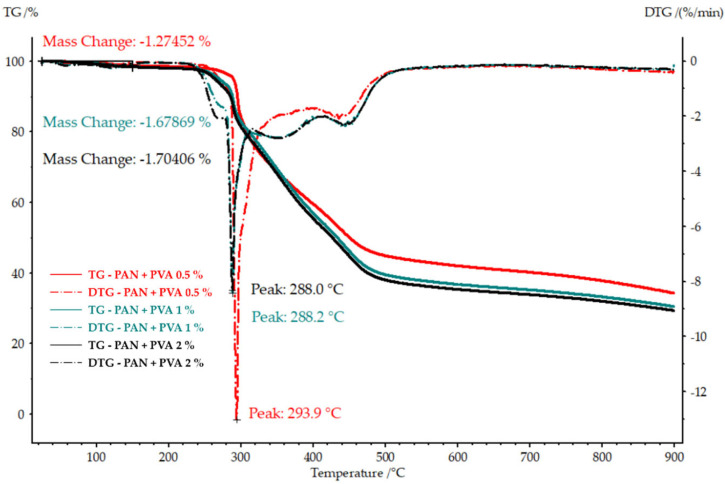
TGA and DTG of the PAN nanofiber mats doped with different concentrations of PVA, illustrating the effect of the PVA concentration on their thermal degradation and early mass loss.

Figure 7 presents the TGA and DTG graphs for the PAN nanofiber mats doped with various concentrations of PVA. In particular, the peak degradation temperature decreases with an increase in the PVA concentration, indicating a shift in the thermal stability of the composite material. Specifically, at a concentration of 0.5% PVA, the peak degradation temperature, 293.9 °C, was similar to that of the undoped PAN nanofiber mat. However, at the 1% and 2% PVA concentrations, the maximum temperatures were lower, 288.2 °C and 288.0 °C, respectively. Conversely, their mass loss up to 150 °C increased with the concentration of PVA, from 1.27% with 0.5% PVA to 1.67% and 1.7% for PVA concentrations of 1% and 2%, respectively.

These observations suggest that as the proportion of PVA in the nanocomposite increases, both its thermal stability (as indicated by its peak degradation temperature) and early mass loss characteristics are impacted. The higher early mass loss in the doped nanofiber mats, although similar to that observed in the PVA film, implies that the presence of PVA influences their thermal behavior. The decrease in peak degradation temperatures with higher PVA concentrations may reflect the inherently lower thermal stability of PVA compared to PAN, affecting the nanofiber mats’ overall thermal resistance. This analysis underscores the interplay between the PVA concentration and the thermal properties of the nanocomposite, offering valuable insights into the material’s behavior under thermal stress.

**Figure 8:**
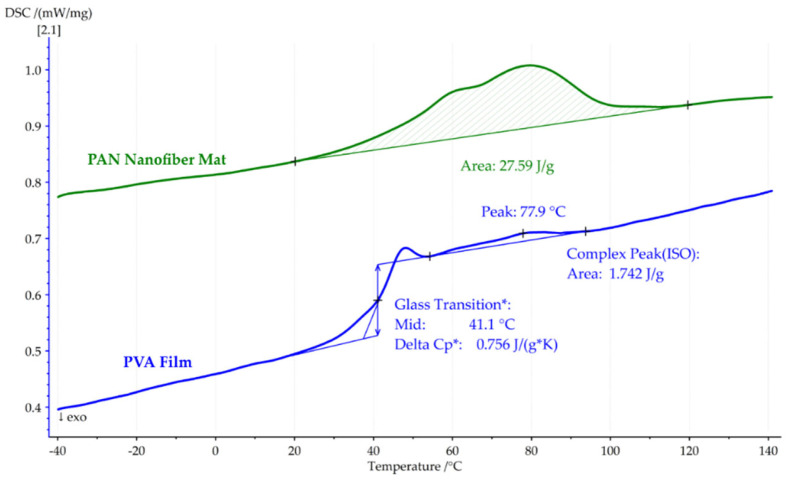
First DSC heating cycle of the pure PAN nanofiber mat and PVA film, showcasing their heat flow and glass transition temperatures.

Figure 8 reveals the results of the first DSC heating cycle for both the pure PAN nanofiber mat and the PVA film. The PAN nanofiber mat showed an absorbed heat of 27.59 J/g, attributed to solvent evaporation, while the PVA film showed a typical glass transition temperature (*Tg*) of 41.1 °C, as well as enthalpy relaxation, a specific heat capacity of ΔCp*: 0.756 J/(g*K), and a mild heat absorption of 1.742 J/g, with a peak at 77.9 °C.

**Figure 9:**
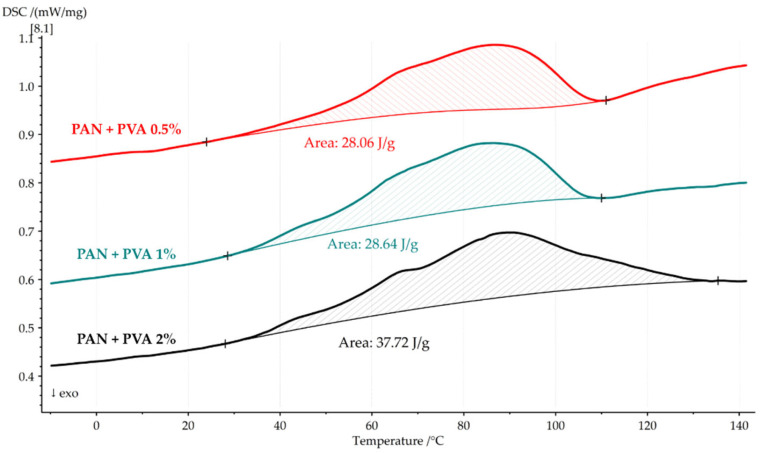
First DSC heating cycle of PAN nanofiber mats doped with PVA, showing their increased heat absorption with higher PVA concentrations.

Figure 9 presents the first DSC heating cycle for the PAN nanofiber mats doped with PVA, indicating that the combined effects of solvent evaporation and glass transition result in their observed heat absorption. For the nanocomposite with 0.5% PVA, the heat flow was 28.06 J/g, increasing to 28.64 J/g and 37.32 J/g for solutions of 1% and 2% PVA, respectively.

This trend suggests that an increasing PVA concentration leads to higher heat absorption, possibly due to the combined effects of solvent evaporation and the relaxation of the enthalpy of PVA within the temperature range of 40 °C to 120 °C.

**Figure 10:**
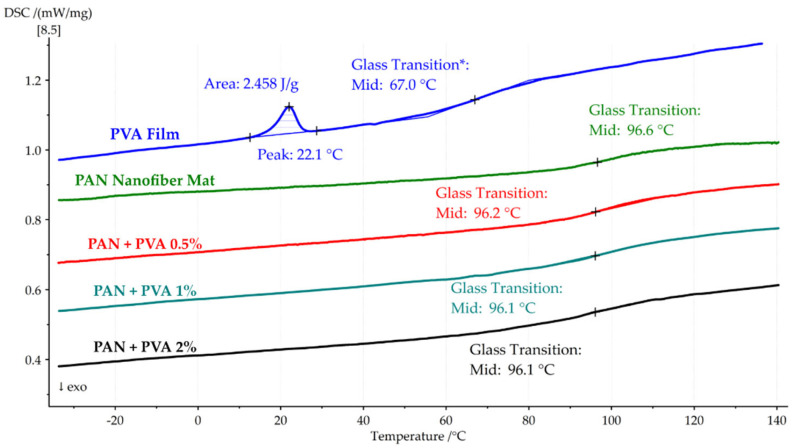
Second DSC heating cycle of the PAN nanofiber mat, the PVA film, and the PAN nanofiber mats doped with PVA, indicating their glass transition temperatures.

In Figure 10, the second DSC heating cycle is shown for the pure PAN nanofiber mat, PVA film, and all composites. The PVA film showed a slight peak at 22 °C, with a heat absorption of 2.45 J/g and a glass transition temperature of 67.0 °C. The glass transition temperature of the PAN nanofiber mat was 96.6 °C, and the doped composites showed similar *Tg* values of 96.2 °C, 96.1 °C, and 96.1 °C for 0.5%, 1%, and 2% PVA, respectively.

Table 3 provides a detailed comparison of the thermal properties, including heat flows and glass transition temperatures, for pure PAN nanofiber mats, PVA films, and PVA-doped PAN nanofiber mats.

This thermal analysis provides a comprehensive understanding of the thermal stability and transitions of the PAN nanofiber mat and its composites. The initial DSC heating cycle highlights the significant role of solvent evaporation in the thermal behavior of the PAN nanofiber mat and demonstrates how the incorporation of PVA influences the heat absorption and glass transition temperatures of the composites. As the PVA concentration increases, a noticeable effect on heat absorption is observed, suggesting enhanced interactions between the PAN nanofibers and PVA which contribute to the thermal behavior of the material. The second heating cycle further elucidates the composites’ glass transition temperatures, indicating that the addition of PVA does not significantly alter the *Tg* of the composites compared to the pure PAN nanofiber mat. These findings not only validate the effectiveness of incorporating PVA into PAN nanofiber mats to potentially improve their thermal properties but also contribute to a broader understanding of this material’s behavior under thermal stress, laying the groundwork for its future use in applications where thermal stability is critical.

### 4.5. Simulation Results

The development of the finite element (FE) model was grounded in previously detailed investigations of the mechanical properties of individual PAN nanofibers [15], with the mechanical properties of PVA incorporated from experimental results obtained in Section 4.3. The elastic response of the structure was evaluated, and the decreased porosity was considered indicative of the volume of dopant within the PAN nanofiber mats. Table 4 compiles all the relevant structural parameters and material properties used to create the model, providing a comprehensive overview to replicate or further explore the discoveries made in this study.

**Figure 11:**
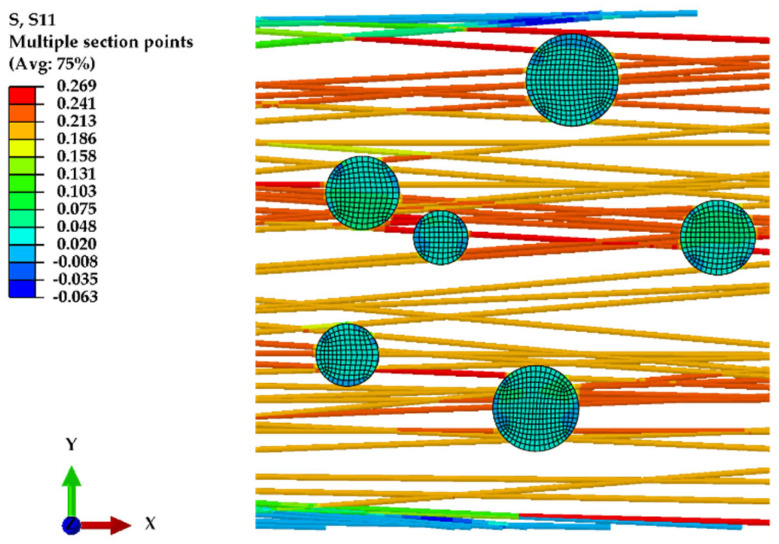
Normal stress on X axis for nanofiber mat doped with 2% PVA.

The FE model depicted in Figure 11 was developed using the parameters outlined in Table 4, illustrating the normal stress distribution, along the X axis, of nanofibers subjected to displacement in the same direction. The introduction of a dopant into the nanofiber mat is shown to enhance its structural rigidity, reinforcing the material’s ability to withstand applied stresses.

**Figure 12:**
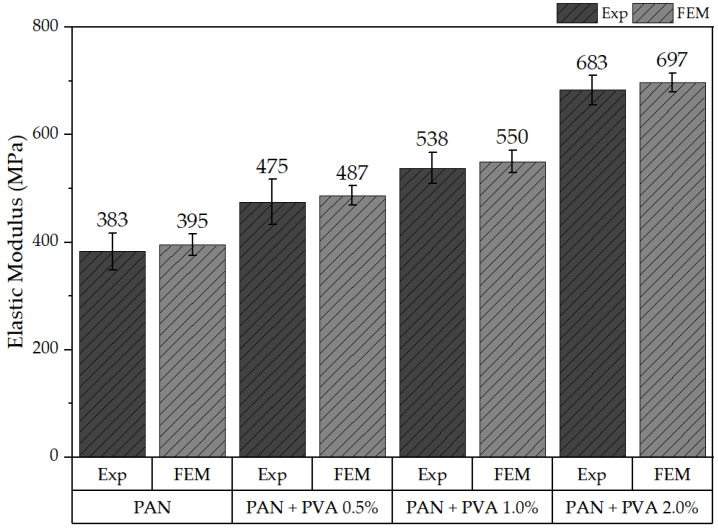
Comparison of the elastic moduli obtained from the experiments and the FE model.

Figure 12 presents the calculated elastic responses of all models alongside the corresponding experimental data, including the undoped PAN nanofiber mat and those doped with PVA at concentrations of 0.5%, 1%, and 2%. In this figure, “Exp” denotes experimental results, while “FEM” represents outcomes derived from the FE model. The results demonstrate that the elastic response of the FE model aligns well with the range of experimental outcomes observed, validating the model’s accuracy. Specifically, the elastic modulus derived from the FE model for the undoped PAN nanofiber mat was found to be 395 MPa ± 19 MPa, while the mats doped with PVA exhibited elastic moduli of 487 ± 18 MPa, 550 ± 21 MPa, and 697 ± 17 MPa, respectively.

Although this research provides valuable information, it acknowledges the limitations inherent to computational modeling. In the model, a crucial assumption was the existence of perfect bonding between the fibers and between fibers and the dopant, effectively overlooking the potential for debonding at the intersections of the fibers and the between the fibers and dopant. The assumption made regarding the volume fraction of PVA dopant within the nanofiber mat, for instance, allows the FE model to closely mimic the experimental outcomes for elasticity. However, the model’s ability to accurately predict plastic behavior, particularly in the presence of a dopant that could form thin films along the fibers, may be less effective. This aspect underscores the complexity of modeling material behavior when the dopant’s distribution and interaction with the matrix material significantly affect the material’s overall structural properties. Future investigations could fruitfully explore these dynamics, potentially refining the model to better capture the nuanced interactions between elasticity, plasticity, and dopant concentration within composite nanofiber mats.

## 5. Conclusions

In this study, we investigated the impact of PVA doping on the mechanical and thermal properties of PAN nanofiber mats through a series of comprehensive experiments and computational modeling. The incorporation of PVA into PAN nanofiber mats via a dip-coating process resulted in significant modifications to the mats’ morphology, including the formation of localized PVA agglomerations, thin PVA films between fibers, and PVA coatings on the fibers. These morphological changes were shown to enhance the structural integrity and mechanical robustness of the nanofiber mats.

Tensile testing revealed that the PVA doping led to an increase in both the elastic modulus and ultimate tensile strength of the nanofiber mats, with the magnitude of improvement correlating to the concentration of PVA used. Specifically, the mats’ longitudinal and transverse mechanical properties exhibited pronounced enhancements compared to the pure PAN nanofiber mat’s properties, suggesting that the PVA dopant effectively reinforced their inter-fiber bonds and provided additional structural support. At a concentration of 2% PVA, the elastic modulus in the longitudinal direction increased by approximately 78.33% and the UTS increased by approximately 84.34%. In the transverse direction, the elastic modulus increased by approximately 159.57%, and the UTS increased by approximately 200.88%.

Thermal analysis, comprising a TGA and DSC, demonstrated that the PVA-doped nanofiber mats exhibited altered thermal degradation behavior and glass transition temperatures, reflecting the influence of PVA on their thermal stability. The observed increase in heat absorption with rising PVA concentration during the DSC heating cycles suggests that the interaction between PAN nanofibers and PVA significantly affects the composite’s thermal behavior, maintaining the glass transition temperatures close to those of the undoped PAN nanofiber mats. During the second heating cycle, a notable increase of 63.02% was observed in the *Tg* of the PVA film. On the contrary, the *Tg* of the composite did not show a significant change in response to the increase in volume of PVA during this cycle.

The FE model developed based on the experimental findings provided further insights into the elastic response of the doped nanofiber mats, confirming the experimental results’ validity. The model highlighted the role of decreased porosity and the presence of the dopant in enhancing the nanofiber mats’ rigidity and elastic properties.

This research highlights the potential of PVA doping to enhance the mechanical and thermal properties of PAN nanofiber mats, offering a promising approach to tailoring the material characteristics of nanofiber-based composites to various applications. Improved mechanical strength and structural integrity, combined with altered thermal properties, make these doped nanofiber mats suitable for use in fields ranging from filtration and tissue engineering to wearable electronics and energy storage.

## Figures and Tables

**Table 1:** Summary of the calculated porosities of the pure and dip-coated PAN nanofiber mats.

Samples	Porosity (%)
PAN Nanofiber mat	72 ± 0.5
PAN + 0.5% PVA	69 ± 0.5
PAN + 1% PVA	67 ± 0.4
PAN + 2% PVA	63 ± 0.7

**Table 2:** Summary of the mechanical properties of the PVA film, PAN nanofiber mat, and PAN nanofiber mats doped with PVA.

Sample	Testing Direction	Thickness, t (µm)	Ultimate Tensile Strength, σ_max_ (MPa)	Young’s Modulus, E (MPa)	Elongation at Break Strain, ε
PVA film	-	53 ± 4	34 ± 2.4	1254 ± 57	0.38 ± 0.03
PAN nanofiber mat	longitudinal	95 ± 7	9.9 ± 0.8	383 ± 34	0.18 ± 0.02
	transverse	97 ± 6	1.13 ± 0.15	47 ± 6	0.21 ± 0.02
PAN + 0.5% PVA	longitudinal	96 ± 5	12.1 ± 1.1	475 ± 42	0.18 ± 0.02
	transverse	98 ± 5	1.87 ± 0.11	65 ± 4	0.16 ± 0.02
PAN + 1% PVA	longitudinal	96 ± 6	14.9 ± 1	538 ± 29	0.23 ± 0.02
	transverse	95 ± 7	2.5 ± 0.1	81 ± 6	0.16 ± 0.02
PAN + 2% PVA	longitudinal	97 ± 4	18.25 ± 1.4	683 ± 27	0.23 ± 0.02
	transverse	101 ± 6	3.4 ± 0.24	122 ± 12	0.14 ± 0.01

**Table 3:** Summary of the thermal properties of the pure PAN nanofiber mat, PVA film, and PAN nanofiber mats doped with PVA, detailing their heat flows and glass transition temperatures.

Properties	Materials				
	PAN	PVA Film	PAN + 0.5% PVA	PAN + 1% PVA	PAN + 2% PVA
DTG peak (°C)	293.3	277.0	293.9	288.2	288.0
Mass loss up to 150 °C (%)	2.43	4.53	1.27	1.67	1.7
Heat absorbed during the first DSC heating cycle (J/g)	27.59	1.742	28.06	28.64	37.32
*Tg* during the first DSC heating cycle (°C)	-	41.1	-	-	-
*Tg* during the second DSC heating cycle (°C)	96.6	67.0	96.2	96.1	96.1

**Table 4:** Summary of the material properties of a single nanofiber and the structural parameters used to develop the FE model.

Structural Parameters and Material Properties	Characteristics/Value
Elastic material model	Hooke’s law
Domain size (µm)	100 × 100
Orientation (°)	+5.5 to −5.5 (11)
Fiber diameter (nm)	610
Fiber length (µm)	10–100.47
Porosity (%)	72
Volume of PVA (%)	9
Doped PVA arrangement	Random
Diameter of PVA dopant (µm)	5 to 10
Elastic modulus fiber (MPa)	2050
Elastic modulus dopant (MPa)	1254
Poisson’s ratio (ν)	0.4
Element size (µm)	1

## Data Availability

Data are contained within the article.
